# Epidemiological Characteristics and Spatial-Temporal Clusters of Hand, Foot, and Mouth Disease in Zhejiang Province, China, 2008-2012

**DOI:** 10.1371/journal.pone.0139109

**Published:** 2015-09-30

**Authors:** Juanjuan Gui, Zhifang Liu, Tianfang Zhang, Qihang Hua, Zhenggang Jiang, Bin Chen, Hua Gu, Huakun Lv, Changzheng Dong

**Affiliations:** 1 Zhejiang Provincial Key Laboratory of Pathophysiology, Department of Preventive Medicine, Ningbo University School of Medicine, Ningbo, Zhejiang Province, China; 2 Zhejiang Provincial Center for Disease Control and Prevention, Hangzhou, Zhejiang Province, China; 3 Women and Children's Hospital of Guangdong Province, Guangzhou, Guangdong Province, China; Kliniken der Stadt Köln gGmbH, GERMANY

## Abstract

Hand, foot and mouth disease (HFMD) is one of the major public health concerns in China. Being the province with high incidence rates of HFMD, the epidemiological features and the spatial-temporal patterns of Zhejiang Province were still unknown. The objective of this study was to investigate the epidemiological characteristics and the high-incidence clusters, as well as explore some potential risk factors. The surveillance data of HFMD during 2008–2012 were collected from the communicable disease surveillance network system of Zhejiang Provincial Center for Disease Control and Prevention. The distributions of age, gender, occupation, season, region, pathogen’s serotype and disease severity were analyzed to describe the epidemiological features of HFMD in Zhejiang Province. Seroprevalence survey for human enterovirus 71 (EV71) in 549 healthy children of Zhejiang Province was also performed, as well as 27 seroprevalence publications between 1997 and 2015 were summarized. The spatial-temporal methods were performed to explore the clusters at county level. Furthermore, pathogens’ serotypes such as EV71 and coxsackievirus A16 (Cox A16) and meteorological factors were analyzed to explore the potential factors associated with the clusters. A total of 454,339 HFMD cases were reported in Zhejiang Province during 2008–2012, including 1688 (0.37%) severe cases. The annual average incidence rate was 172.98 per 100,000 (ranged from 72.61 to 270.04). The male-to-female ratio for mild cases was around 1.64:1, and up to 1.87:1 for severe cases. Of the total cases, children aged under three years old and under five years old accounted for almost 60% and 90%, respectively. Among all enteroviruses, the predominant serotype was EV71 (49.70%), followed by Cox A16 (26.05%) and other enteroviruses (24.24%) for mild cases. In severe cases, EV71 (82.85%) was the major causative agent. EV71 seroprevalence survey in healthy children confirmed that occult infection was common in children. Furthermore, literature summary for 26 seroprevalence studies during 1997–2015 confirmed that 0–5 years group showed lowest level of EV71 seroprevalence (29.1% on average) compared to the elder children (6–10 years group: 54.6%; 11–20 years group: 61.8%). Global positive spatial autocorrelation patterns (Moran’s *I*s>0.25, *P*<0.05) were discovered not only for mild cases but also for severe cases, and local positive spatial autocorrelation patterns were revealed for counties from the eastern coastal and southern regions. The retrospective space-time cluster analysis also confirmed these patterns. Risk factors analyses implied that more EV71 and less sunshine were associated with the clusters of HFMD in Zhejiang Province. Our study confirmed that Zhejiang Province was one of the highly epidemic provinces in China and that the epidemiological characteristics of HFMD were similar to other provinces. Occult infection in elder children and adults was one of the important reasons why most HFMD cases were children aged under-five. Combining the results of spatial autocorrelation analysis and the space-time cluster analysis, the major spatial-temporal clusters were from the eastern coastal and southern regions. The distribution of pathogens’ serotypes and the level of sunshine could be risk factors for, and serve as an early warning of, the outbreak of HFMD in Zhejiang Province.

## Introduction

Hand, foot, and mouth disease (HFMD) is a common enteroviral infectious disease which is mainly caused by the infection of human enterovirus 71 (EV71) and coxsackievirus A16 (Cox A16) [1–3]. Young children, especially those under the age of five years (under-five), are the group most susceptible to HFMD [4–6]. HFMD is generally mild and self-limiting with common symptoms such as fever, rashes or herpes appearing on the hands, feet or mouth. However, complications (such as meningitis, encephalitis and pulmonary edema) mainly caused by the infection of EV71 can lead to severe cases and even deaths, especially for young children [7, 8]. Since enteroviruses have high infectivity and can transmit through different ways such as fecal-oral channel, respiratory tract, or touching contaminants, it’s rather easy to have outbreaks in nursery centers or schools where the high density of young children contributes to the transmission of enteroviruses. Therefore, it’s still a great challenge to prevent and control the outbreak of HFMD given the lack of effective drugs or vaccine [9–11].

The first record of HFMD was reported in New Zealand and Canada in 1957 [12, 13] and then HFMD was majorly prevalent in the West [14–16]. Since 1997, several great outbreaks of HFMD emerged in the Asia-Pacific region, especially: Malaysia [17, 18], Taiwan [19, 20] and Australia[21]. As for mainland China, the first case of HFMD was reported in the Shanghai Municipality in 1981 [22], and then some small outbreaks occurred in the Tianjin Municipality [22] and Shandong Province [23]. After its great outbreak at Fuyang of Anhui Province, which caused 22 deaths during three months [24], HFMD was under surveillance by the communicable disease surveillance network system of the China Center for Disease Control and Prevention (CDC). In recent years, outbreaks of HFMD were still common in most of the provinces of China and threatened the health of young children. For example, Shandong Province [5, 23], Guangdong Province [25, 26] and Beijing Municipality [6] had reported that boys under-five were the most susceptible group to HFMD and spatial-temporal patterns steadily occurred.

**Fig 1:**
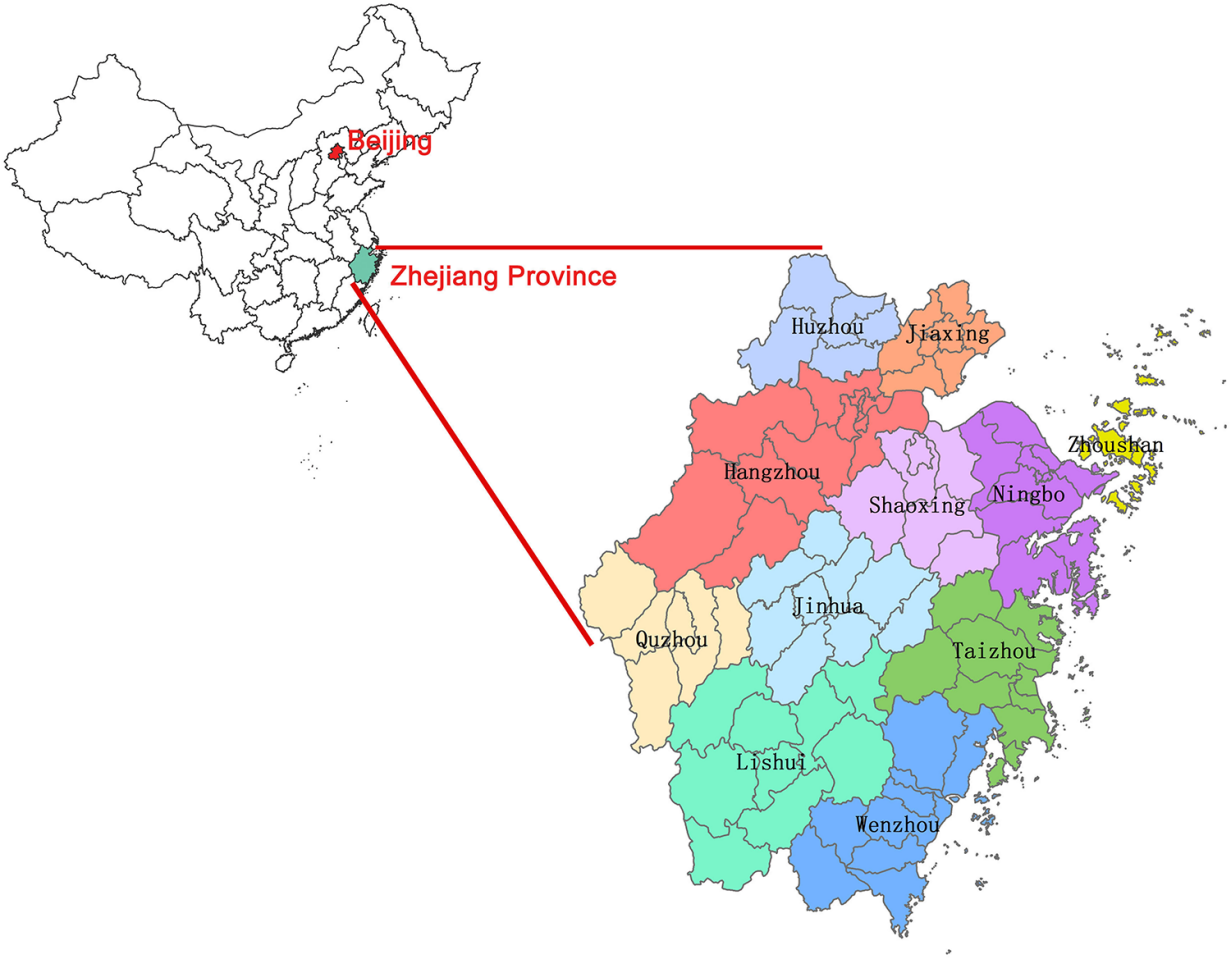
The map of Zhejiang Province.

Zhejiang Province, with a geographical location between 27.21N to 31.52N and 118E to 123E, is one of the eastern coastal provinces of China (Fig 1). It has a population of approximately 54.77 million and covers an area of 101.8 thousand square kilometers with 11 districts comprising 90 counties in total. As a province with a subtropical monsoon climate, plentiful rainfall and high humidity make it easier for the reproduction and transmission of enteroviruses. Being one of the provinces with high incidence rates of HFMD in China, Zhejiang Province had an annual incidence rate of about 150 per 100,000 in the total population in recent years, and more than one thousand severe cases with all kinds of complications were reported in 2010 [27].

Therefore, we analyzed the epidemiological characteristics and performed the spatial-temporal analysis based on the surveillance data of Zhejiang Province, 2008–2012 to discover the potential clustered regions of HFMD which were important for the control and prevention of HFMD. Pathogens’ serotypes, seroprevalence and meteorological factors were analyzed to explore the potential factors associated with the clusters.

## Materials and Methods

### Data collection

The surveillance data of HFMD from 2008 to 2012 were collected from the communicable disease surveillance network system of the CDC of Zhejiang Province, including each patient’s disease related demographic information such as gender, occupation, age, date of diagnosis and disease severity (mild case or severe case). The pathogens’ serotypes (EV71, Cox A16 or other enteroviruses) identified by the real-time fluorescence quantitative polymerase chain reaction (qRT-PCR) were available for part of patients. Demographic information of 90 counties was provided by the Zhejiang Provincial Statistics Bureau. Meteorological data including monthly average temperature, monthly average rainfall and monthly average sunshine of 11 districts were collected from the Zhejiang Statistical Yearbook.

### Ethics statement

This retrospective study was approved by the Ethics Committee of Ningbo University School of Medicine and Zhejiang Provincial Center for Disease Control and Prevention. Since all analyses were at least county-based, all individual identifying information (including name, address and telephone, etc) was anonymized and de-identified prior to analysis. All individuals participating in the serological study signed informed consent forms by themselves or their parents/guardian. The study was carried out in a manner conforming to the Declaration of Helsinki.

### Basic epidemiological and statistical analysis

Descriptive statistics (incidence rates, distributions of age, gender, occupation, year and month, pathogen’s serotype and disease severity) were used to describe the epidemiological characteristics of HFMD annually. The correlation between the proportion of EV71 or Cox A16 among all enteroviruses and the case-severity rate (the rate of the severe cases among total cases) were assessed by the partial correlation coefficients. Meteorological factors (monthly average temperature, monthly average rainfall and monthly average sunshine) from April to July (the annual peak of the incidence) were compared between cases-clustered regions and non-clustered regions with student *t* tests. Chi-square tests were used to compare HFMD’s distributions of ages and pathogens’ serotypes between mild and severe cases. These statistical analyses were performed using SPSS 17.0 (http://www-01.ibm.com/software/analytics/spss/).

### Age-specific EV71 seroprevalence survey and summary in healthy children

Participants were selected by stratified random sampling from four districts (Hangzhou, Ningbo, Taizhou and Jinhua) of Zhejiang Province. The sample sizes for each age-group (0–5, 6–10, 11–20) were calculated according to the age-specific incidence rates of HFMD in Zhejiang Province. Non-HFMD children in pediatric infectious wards were randomly chosen and those who had HFMD histories or rejected to subscribe names in the written informed consent forms were excluded. Five ml venous bloody samples were collected from each participant, then serum were separated and stored at -70°C until testing. All specimens were tested for human antibody immunoglobulin G to EV71 (EV71-IgG) using Enzyme-Linked Immunosorbent Assay (ELISA) with the criterion that an S/N≥2.1 was considered to be positive. ELISA steps were completed according to the guidance of reagent kids which were registered by China Food and Drug Administration and produced by Beijing Beier Bioengineering CO., LTD.

To summarize the age-specific EV71 seroprevalence in healthy children since 1997, publications from 1997 to 2015 were retrieved from NCBI Pubmed and Google Scholar by Jun 30, 2015. The keywords included “human enterovirus 71” or “enterovirus 71” or “EV 71”, together with “seroprevalence” or “seroepidemiology” or “serological” or “seroincidence” or “antibody” or “neutralizing antibody” or “microneutralization test” or “IgG antibody” or “ELISA” or “serum samples (or sera)” or “geometric mean titer (GMT)”. Our literature search harvested a total of 27 publications. Among them, four HFMD cases based publications [28–31], one adult based [32] and one study without detailed data [33] were excluded. Thus, the age-specific EV71 seroprevalence summary was conducted on 21 publications [34–54] and our study.

### Spatial autocorrelation analysis

As a spatial method used to analyze the spatial autocorrelation association based on the locations of study regions, global Moran’s *I* [55] was used to detect whether the significant spatial autocorrelation regions of HFMD in Zhejiang Province existed. If so, then local Moran’s *I* was further used to clarify the patterns of spatial autocorrelation among local counties. The significance of Moran’s *I* was validated by Monte Carlo tests with *Z* statistics and the *P* values. Positive spatial autocorrelation (high-incidence clusters or low-incidence clusters) is exhibited if Moran’s *I* is larger than zero with the statistical significance, while negative autocorrelation (mixed clusters with both high-incidence and low-incidence regions) for negative *I*. By using local indicators of spatial association (LISA) map [56–58], four patterns of spatial correlation with high-high (high-incidence regions surrounded by high-incidence regions, which are highly epidemical regions), low-low (low-incidence regions surrounded by low-incidence regions, which are lowly epidemical regions), high-low (high-incidence regions surrounded by low-incidence regions) and low-high (low-incidence regions surrounded by high-incidence regions) were demonstrated. In reality, the high-high pattern or so-called hot spot is most useful for disease control and prevention. The spatial weights used to describe the spatial relationships among counties in Zhejiang Province were created by queen contiguity rule and Geoda (V1.4.0) software (http://geodacenter.asu.edu/projects/opengeoda) was used to perform the above analyses.

The formula of global Moran’s *I* was listed as follow:

$$I=\frac{n\sum_{i=1}^{n}\sum_{j=1}^{n}w_{ij}(x_{i}-\bar{x})(x_{j}-\bar{x})}{\sum_{i=1}^{n}\sum_{j=1}^{n}w_{ij}{\left(x_{i}-\bar{x}\right)}^{2}}$$

Where *n* is the number of districts, *i* and *j* are two different districts, *x*_*i*_ and *x*_*j*_ are the values of the observed indicators (such as incidence rate) for district *i* and *j*, $\bar{x}$ is the average of the indicator of all districts, and *w*_*ij*_ is the spatial weight indicator. For local Moran’s *I*, the formula is the same except that *i* and *j* refer to the local counties.

### Space-time cluster analysis

Based on a moving cylindrical window with dynamic changes of circular base and height corresponding to space and time respectively, the space-time scan statistic can be used to detect possible spatial-temporal clusters by producing an infinite number of overlapping cylinders with different radiuses [59, 60]. For each window, the expected number of cases can be inferred by using the discrete Poisson model or Bernoulli model with the observed number of cases and the number of the population within/outside the moved windows (the potential clusters) of candidate regions during candidate time. The relative risk (RR) can be calculated by the ratio of the observed number to the expected number within the windows and outside the windows, as well as the log likelihood ratio (LLR) is calculated by a likelihood function. The most likely cluster is the one with the maximum LLR and the significance of it can be inferred through Monte Carlo tests with 1,000 permutations.

For each space-time scanning window, the alternative hypothesis is that there is an elevated risk within the window as compared to the outside. The likelihood ratio (LR) according to the discrete Poisson model can be calculated as follows:

$$LR=\frac{L(Z)}{L_{0}}={\left(\frac{c}{\mu (Z)}\right)}^{c}{\left(\frac{C-c}{C-\mu (Z)}\right)}^{C-c}$$

Where *C* is the total number of cases, *c* is the observed number of cases within the window *Z*, *μ*(Z) is the expected number of cases within the window under the null-hypothesis, and *C*−*c* and *C*−*μ*(*Z*) are the observed and expected numbers of cases outside the window.

In our study, the retrospective space-time statistic was used to analyze the spatial-temporal clusters of the collected data. It was specified that the maximum spatial size of the clusters is 20% of the total population at risk and the maximum temporal size of the clusters is 50% of the study period. The cluster with maximum LLR was regarded as the most likely cluster while others that also have significant *P*-values were named as secondary clusters. The space-time cluster analysis was performed using SatScan 9.3 (http://www.satscan.org/).

### Phylogenetic analysis

A phylogenetic tree for the VP1 gene of EV71 was constructed to show the phylogenetic relationships of EV71 strains isolated from China. Details of material and methods can be seen in supplemental material and methods (S1 File).

## Results

### Epidemiological characteristics

A total of 454,339 HFMD cases were reported in Zhejiang Province, 2008–2012 with the average annual incidence rate of 172.98 per 100,000 (range from 72.61 to 270.04, S1 Fig). Of 454,339 HFMD cases, 1688 (0.37%) were severe cases (most were encephalitis or meningitis). Since males are more susceptible to HFMD, the male-to-female ratio for mild cases is around 1.64:1 while up to 1.87:1 for severe cases (Table 1). Of the total cases, children aged under-three and under-five accounted for almost 60% and 90%, respectively. A significant difference (*χ* ^2^ = 154.92, *P*<0.001) had been found between the age distributions of mild cases and severe cases where more severe cases (74.05%) occurred in the under-three group. Corresponding to the age distribution, children scattered at home accounted for about 70% and 85% of mild cases and severe cases respectively, followed by nursery children (27.68% and 13.39%) and school students (2.37% and 1.30%) respectively.

**Table 1:** Epidemiological characteristics of HFMD cases from Zhejiang Province, 2008–2012.

Cases type	Year	Sex ratio (M:F)	Age (years)			Occupation (Children)*			Pathogen’s serotype		
			0–3 (%)	3–5 (%)	>5 (%)	Scattered (%)	Nursery (%)	School (%)	EV71 (%)	Cox A16 (%)	Others (%)
Mild cases	2008	1.70:1	60.69	27.62	11.69	68.83	27.49	3.11	54.58	4.37	41.05
	2009	1.67:1	62.53	28.84	8.63	71.71	25.92	1.99	41.73	40.04	18.24
	2010	1.64:1	58.32	29.88	11.79	68.94	28.45	2.33	54.67	30.36	14.97
	2011	1.65:1	60.25	29.84	9.91	69.92	27.46	2.19	56.14	20.11	23.75
	2012	1.60:1	57.45	31.40	11.15	69.06	28.08	2.49	39.68	30.57	29.75
	Total	1.64:1	59.24	30.03	10.73	69.58	27.68	2.37	49.70	26.05	24.24
Severe cases	2008	1.60:1	74.39	20.12	5.49	83.54	15.24	1.22	69.05	0.00	30.95
	2009	1.98:1	78.52	19.46	2.01	88.59	8.72	2.01	71.03	19.63	9.35
	2010	1.97:1	73.94	20.73	5.33	87.86	10.86	1.28	87.71	2.43	9.86
	2011	1.76:1	72.11	22.71	5.18	76.10	21.51	1.59	82.49	1.38	16.13
	2012	1.52:1	72.97	21.62	5.41	78.38	21.62	0.00	73.68	7.89	18.42
	Total	1.87:1	74.05	20.91	5.04	85.13	13.39	1.30	82.85	4.02	13.12

*Since most cases were children (<18), only these children’s occupations (scattered, nursery or school) were classified.

**Fig 2:**
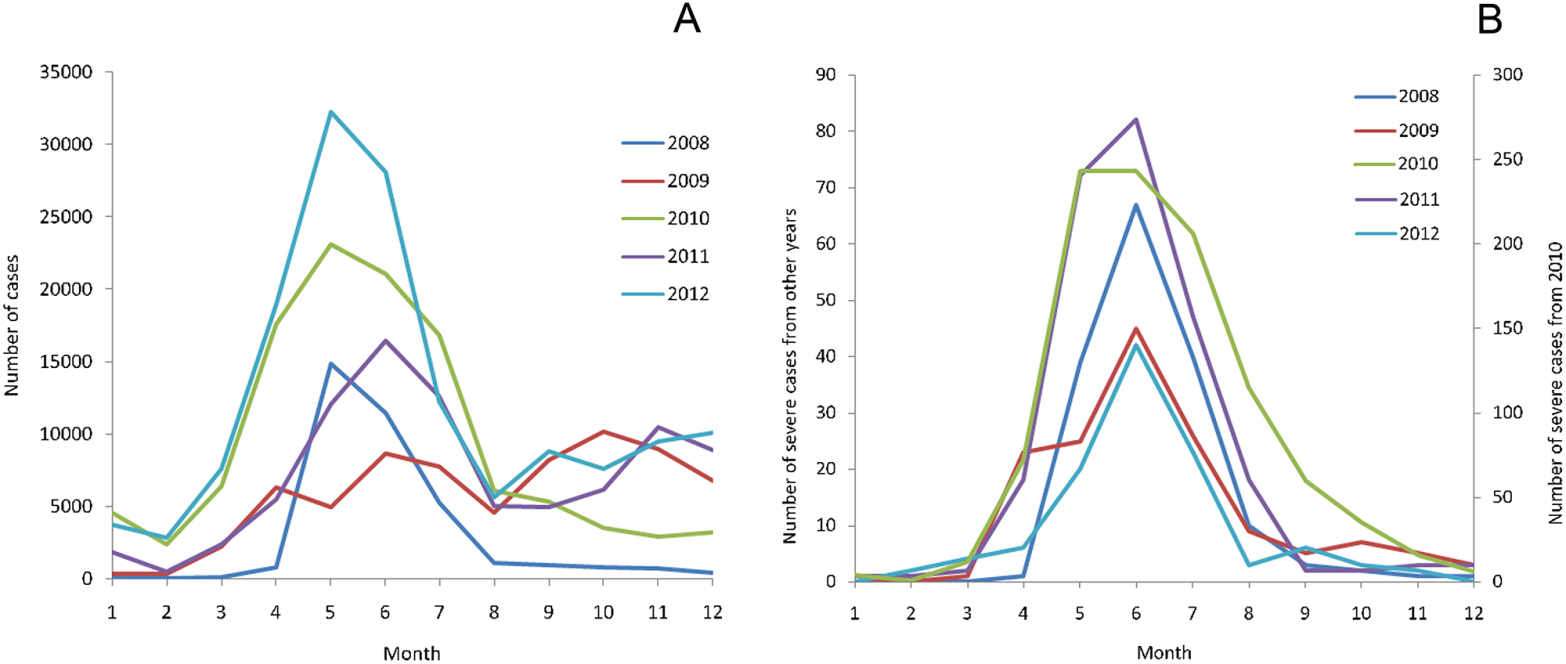
The monthly distribution of the number of mild cases (A) and severe cases (B) during 2008–2012.

**Fig 3:**
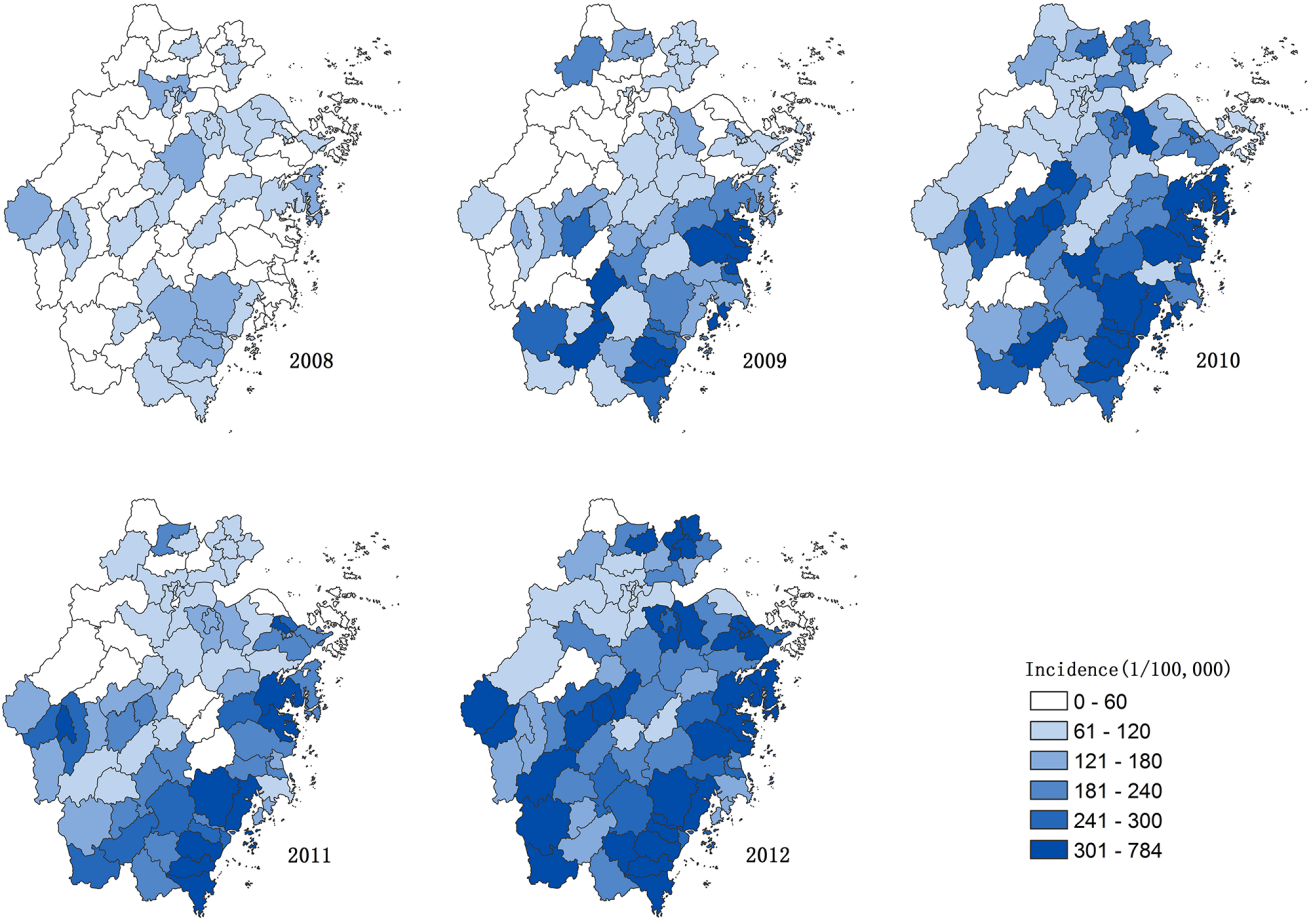
The incidence rates of counties in Zhejiang Province, 2008–2012.

The variation of monthly distribution of HFMD was shown in Fig 2, which indicated that the number of cases came to arise from March (13^rd^ week) and reached the first and highest peak between April and July (Fig 2A). September (36^th^ week) was the start of the second peak observed between October and December. As seen with severe cases, a clear peak was only observed less than a month following the appearance of the first peak of mild cases (Fig 2B). In 2010, a large outbreak of severe cases in Wenzhou caused an especially high peak for that season (Fig 2B and S1 Fig). When Wenzhou was excluded annually, the number of severe cases of Zhejiang Province in 2010 is similar to those of other years (S2 Fig). The variation of geographical distribution of HFMD of mild and severe cases can be observed from Fig 3 and S3 Fig, indicating that five districts (Wenzhou, Taizhou, Lishui, Ningbo and Quzhou) had higher incidences than others (Fig 3) and the districts with the top incidences of severe cases were Wenzhou, Quzhou, Ningbo and Lishui (S2 Fig). In a word, the eastern coastal and southern counties were the major regions with high incidences of HFMD while the northwest and the central regions were the relatively low-incidence regions.

### Distributions of pathogens’ serotypes

**Fig 4:**
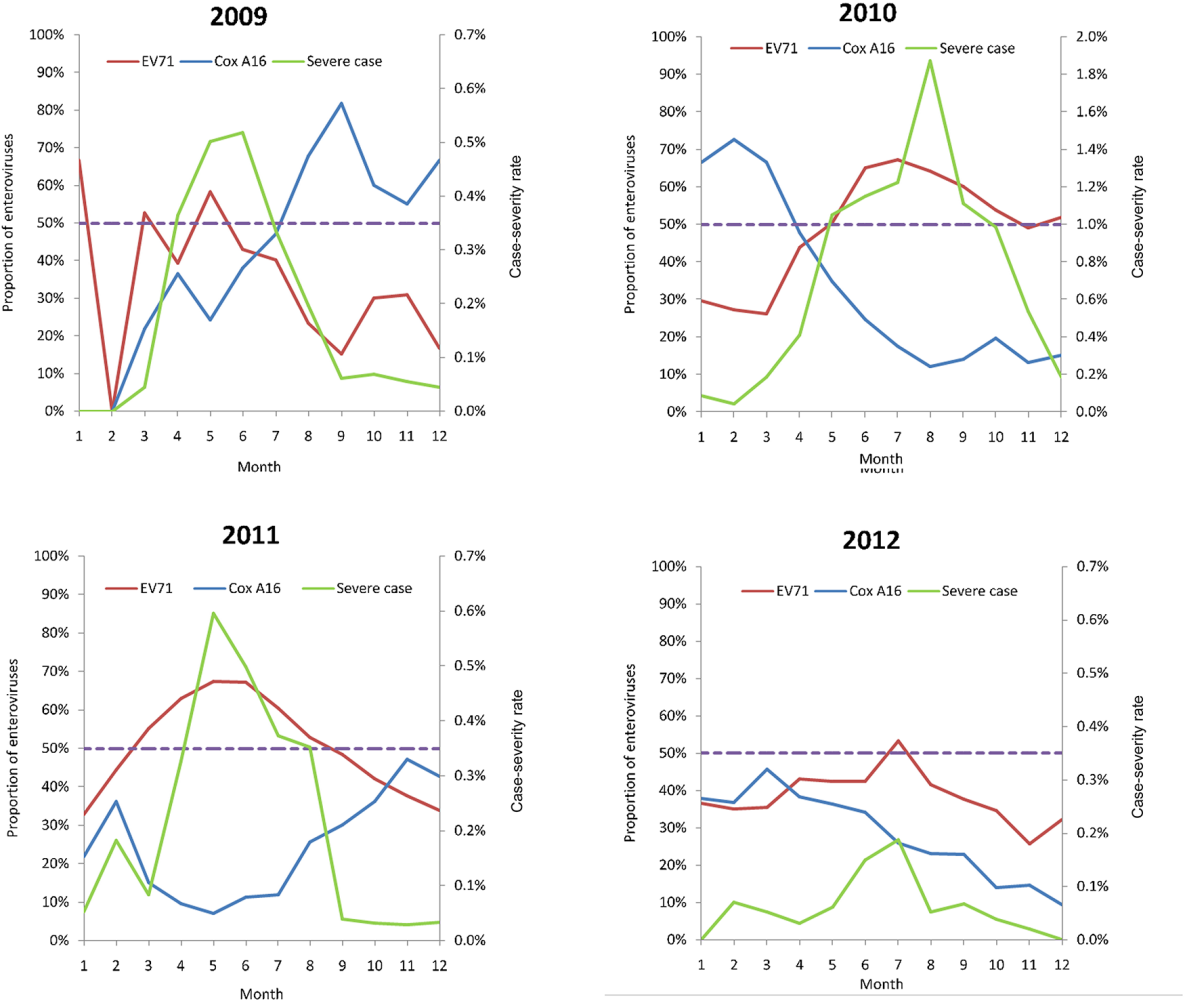
The partial correlations between the monthly proportions of EV71/Cox A16 and the case-severity rate.

Of 454,339 cases, 15,055 (3.31%) were laboratory-confirmed cases including 1143 severe cases. The predominant serotype was EV71 (accounting for 49.70%), followed by Cox A16 (26.05%) and other enteroviruses (24.24%) for mild cases (Table 1). The distribution of pathogens’ serotypes of severe cases was different from that of mild cases (*χ* ^2^ = 566.89, *P*<0.001) where EV71 (82.85%) is the major causative agent. The monthly distributions of the composition of the enteroviruses’ serotypes are shown in S4 Fig. It was clear that EV71 and Cox A16 were almost the most important pathogens in each month. The positive correlation (partial correlation coefficient = 0.621, *P*<0.001) between the monthly proportion of EV71 and the case-severity rate was observed when time factors (year and month) were controlled (Fig 4). The negative correlation (partial correlation coefficient = -0.325, *P* = 0.028) between Cox A16 and the case-severity rate was also observed. When the proportions of EV71 exceeded 50%, the rates of severe cases reached their peaks except for 2010. In this year, the peak of severe cases was delayed and occurred in autumn. Since most of the severe cases of 2010 were from Wenzhou, we excluded the cases from Wenzhou annually. The positive correlation between EV71 and severe cases, and the negative correlation between Cox A16 and severe cases, were also observed for each year including 2010 (S5 Fig). Furthermore, the fact that 80% of pathogens’ serotypes of all cases were EV71 in Wenzhou and the proportion was much higher than other districts in Zhejiang Province, may hint at the cause of the outbreak of severe cases in Wenzhou. According to that, there were fewer cases of EV71 in its peak in 2012; there were also fewer severe cases in that year (Fig 4 and S1 Fig). Since data for pathogens’ serotypes were unreliable for several months in 2008 (S4 Fig), we did not show the correlation graph for 2008 in Fig 4 and S5 Fig.

### Age-specific EV71 seroprevalence survey and summary in healthy children

To test the hypothesis that the relatively low level of EV71 antibody was the reason for high HFMD incidence in children aged under-five, age-specific EV71 seroprevalence was surveyed in healthy children in Zhejiang Province via ELISA. In total, 49.9% (274/549) healthy children showed positive EV71-IgG (Table 2) which indicated occult infection was common in children. To our surprise, the seroprevalence of the young children (0–5 years: 53.0%) was higher than those of elder children (6–10 years: 42.3%; 11–20 years: 30.8%). Thus, age-specific EV71 seroprevalence summary in healthy children since 1997 was performed to describe the age distribution of EV71 seroprevalence in healthy children and verify the above hypothesis.

**Table 2:** Age-specific EV71 seroprevalence (EV71-IgG) in healthy children of Zhejiang Province, China.

Age group (years)	No. samples	No. Positive	Positive rate (%)	95% CI (%)
0–5	445	236	53.0	(48.4, 57.6)
6–10	52	22	42.3	(28.9, 55.7)
11–20	52	16	30.8	(18.3, 43.3)
Total	549	274	49.9	(45.7, 54.1)

**Fig 5:**
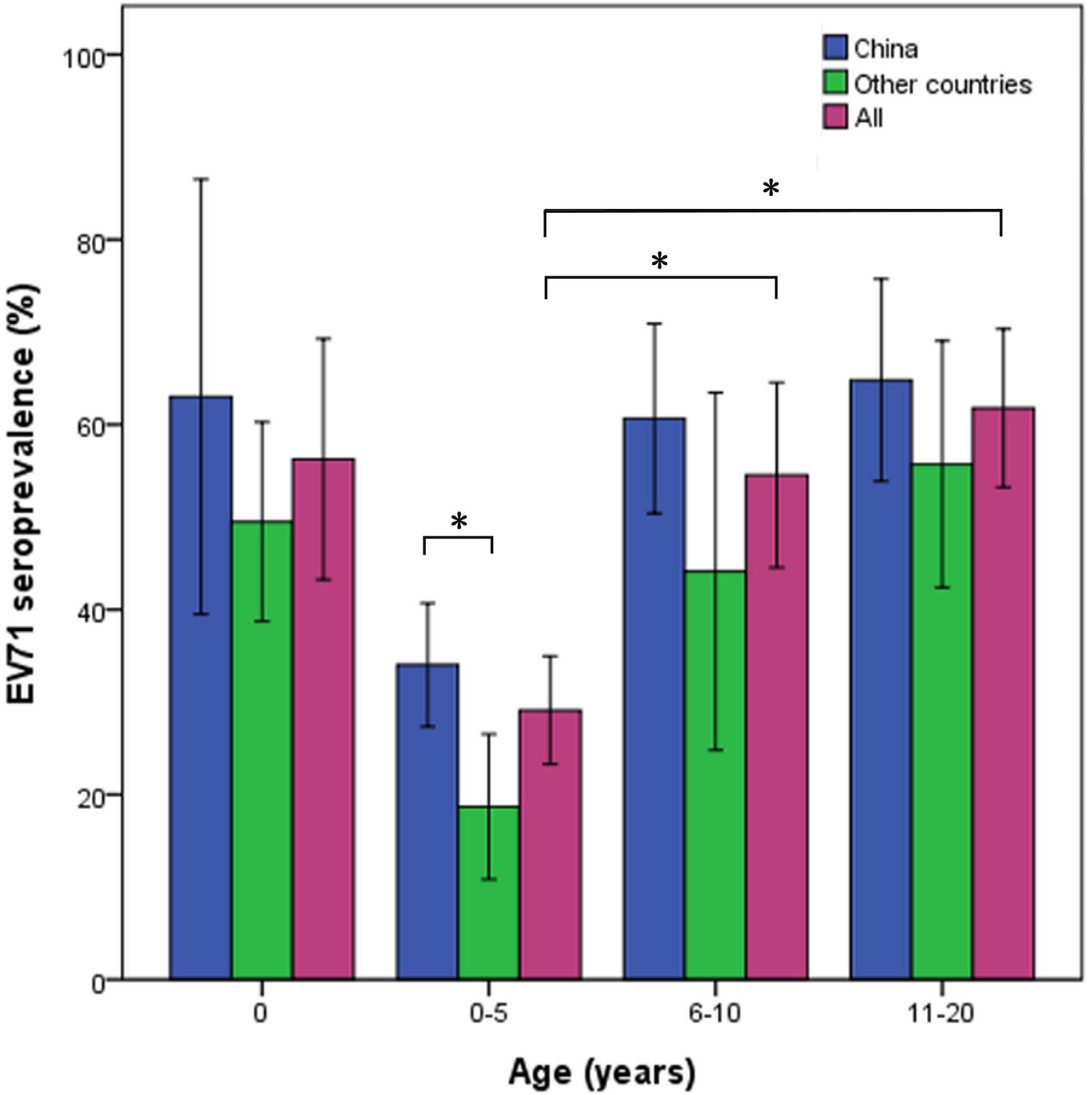
Age-specific EV71 seroprevalence summary in healthy children.

Among 26 seroprevalence studies (pre- and post-epidemic studies were performed in four publications) during 1997–2015, two-thirds were surveyed in mainland China and Taiwan (Table 3). Almost all studies used neutralizing antibody to test serum antibodies except for our and Kuang et al.’s studies where EV71-IgG was detected by ELISA. Only four studies were based on neonates (0 year) and cord blood was used instead of venous blood. The neonates exhibited high seroprevalence (44.0–75.0%) which implied that their mothers (the source of the neonates’ antibodies) had high level of EV71 antibody. In general, the level of the neonates’ antibodies declined until six months after birth when occult infection could induce the active immunity. Therefore, 0–5 years group (children aged under-five) showed lowest level of EV71 seroprevalence (29.1% on average, Table 3 and Fig 5). Since the elder children had more chances of exposure to the enteroviruses and repeated active immunity, their seorprevalences were significant higher than the younger (6–10 years group: 54.6%, *t* = 4.540, *P*<0.001; 11–20 years group: 61.8%, *t* = 6.406, *P*<0.001). This age-specific EV71 seroprevalence pattern provided the evidence that children aged under-five were most susceptible to HFMD. Another hypothesis was that HFMD epidemics would significantly increase the level of EV71 seroprevalence after the epidemics. However, the results of four studies were contradictory (Table 3): one positive result (Study ID: 9&10) and three mixed results (Study ID: 5&6, 15&16, 17&18). Furthermore, as a country confronting the long-term exposure to HFMD epidemics, it was considered that China may have a higher level of seroprevalence than other counties. Fig 5 indicated that only 0–5 years group exhibited significant difference between China and other counties (*t* = 2.694, *P* = 0.013) which implied the complex relationship between the seroprevalence and the history of exposure.

**Table 3:** Summary of the EV71 seroprevalence studies in healthy children since 1997.

ID	Sampling year^a^	Author	Region	Population^b^	Tested Antibody^c^	Sample size					Seroprevalence (%)				
						0 yr^d^	0–5 yrs	6–10 yrs	11–20 yrs	Total	0 yr^d^	0–5 yrs	6–10 yrs	11–20 yrs	Total
1	2014	This study	China	S, H	I	-	445	52	52	549	-	53.0	42.3	30.8	49.9
2	2011	Ni et al.[49]	China	O	N	-	147	37	37	221	-	34.0	67.6	86.5	48.4
3	2010–2011	Zeng et al.[50]	China	O	N	-	614	-	-	614	-	19.9	-	-	19.9
4	2010	Kuang et al.[44]	China	S	I	-	652	167^e^	167^e^	819	-	30.8	22.8^e^	22.8^e^	29.2
5	2010(a)	Li et al.[53]	China	S	N	-	280	50^e^	50^e^	330	-	52.7	78.0^e^	78.0^e^	56.5
6	2010(b)	Li et al.[53]	China	S	N	-	194	98^e^	98^e^	292	-	64.1	71.0^e^	71.0^e^	66.4
7	2010	Ji et al.[48]	China	S	N	40	640	80	80	840	75.0	34.7	92.0	80.0	46.4
8	2007–2010	Zhu et al.[51]	China	C	N	-	975	-	-	975	-	49.3	-	-	49.3
9	2006–2010(a)	Yu et al.[47]	China	S	N	-	49	25	9	83	-	36.7	72.0	77.8	51.8
10	2006–2010(b)	Yu et al.[47]	China	S	N	-	235	158	79	472	-	24.3	55.7	74.6	43.2
11	2008	Yang et al.[46]	China	S	N	-	60	-	-	60	-	41.2	-	-	41.2
12	2006–2008	Luo et al.[38]	Taiwan	C	N	459	-	-	-	459	51.0	-	-	-	51.0
13	2006–2007	Huang et al.[43]	Taiwan	C	N	-	287	-	-	287	-	12.5	-	-	12.5
14	2005	Zhu et al.[41]	China	S	N	-	900	-	-	900	-	32.0	-	-	32.0
15	1997–1999(a)	Chang et al.[34]	Taiwan	O	N	-	2091	761	648	3500	-	24.8	61.0	65.0	40.1
16	1997–1999(b)	Chang et al.[34]	Taiwan	O	N	-	216	48	79	343	-	20.2	63.0	66.0	36.8
17	1994–1999(a)	Lu et al.[35]	Taiwan	O	N	-	576	108	264	948	-	23.3	45.4	63.0	36.9
18	1994–1999(b)	Lu et al.[35]	Taiwan	O	N	-	281	54	64	399	-	24.9	57.0	62.3	35.2
19	2008–2010	Ang et al.[42]	Singapore	S	N	-	327	290	566	1183	-	13.2	22.9	37.9	26.9
20	1994–2010	Honkanen et al.[52]	Finland	O	N	-	505^e^	505^e^	-	505	-	1.6^e^	1.6^e^	-	1.6
21	2005–2009	Tran et al.[45]	Viet Nam	S	N	200	1194	120^e^	120^e^	1514	55.0	11.0	84.0^e^	84.0^e^	24.6
22	2007–2008	Akhmadishina et al.[54]	Russia	O	N	-	826	-	-	826	-	29.2	-	-	29.2
23	1997–2007	Diedrich et al.[37]	Germany	S	N	-	112	55	109	276	-	36.6	58.0	64.6	51.7
24	2006	Rabenau et al.[40]	Germany	O	N	-	100	100	200	400	-	12.0	47.0	51.0	40.3
25	2004	Mizuta et al.[39]	Japan	O	N	-	29	22	7	58	-	24.1	50.0	42.9	36.2
26	1996–1997	Ooi et al.[36]	Singapore	S	N	70	463	238	155	926	44.0	21.9	45.3	53.9	34.9

^a^: after the epidemic (a); before the epidemic (b). For example, 2010(a) and 2010(b) are the seroprevalence results after and before the 2010 HFMD epidemic.

^b^: S: healthy children defined as "no HFMD symptom or no sign at the time of the survey"; H: no HFMD history; O: serum samples collected from studies for other diseases or general population; C: HFMD cohort study.

^c^:IgG antibody (I); Neutralizing antibody (N).

^d^:0 yr are neonates (cord blood).

^e^:In original paper, the nearby age groups are merged.

### Spatial autocorrelation analysis

Based on the surveillance data of counties of Zhejiang Province, 2008–2012, globally positive spatial autocorrelation association (Moran’s *I* ranged from 0.29 to 0.47 with statistical significances) for mild cases was revealed by using the global spatial autocorrelation analysis which indicated the nonrandom distribution of HFMD in Zhejiang Province (Table 4). The LISA maps were used to illustrate the results of local spatial autocorrelation analysis (S6 Fig). It shows that high-incidence clusters (high-high pattern, dark red color) were mostly from eastern coastal and southern districts including Wenzhou, Lishui, Taizhou, Ningbo and Quzhou while northwest of Zhejiang Province were low-incidence regions (low-low pattern, dark blue color). Other districts were mixed clusters or non-cluster regions. According to severe cases, Moran’s *I*s were higher (range from 0.25 to 0.67) especially for 2010 (S1 Table). At first glance, the high-incidence regions of severe cases were similar to those of mild cases (S7 Fig). Even though, Ningbo was a major difference for severe cases and showed a high-high pattern in the LISA map for several years.

**Table 4:** The Moran’s *I* of global spatial autocorrelation analysis for mild cases of HFMD during 2008–2012.

Year	Moran's *I*	*Z* score	*P*-value
2008	0.29	5.43	<0.001
2009	0.41	6.83	<0.001
2010	0.35	6.12	<0.001
2011	0.47	8.90	<0.001
2012	0.41	6.25	<0.001

### Space-time cluster analysis

**Fig 6:**
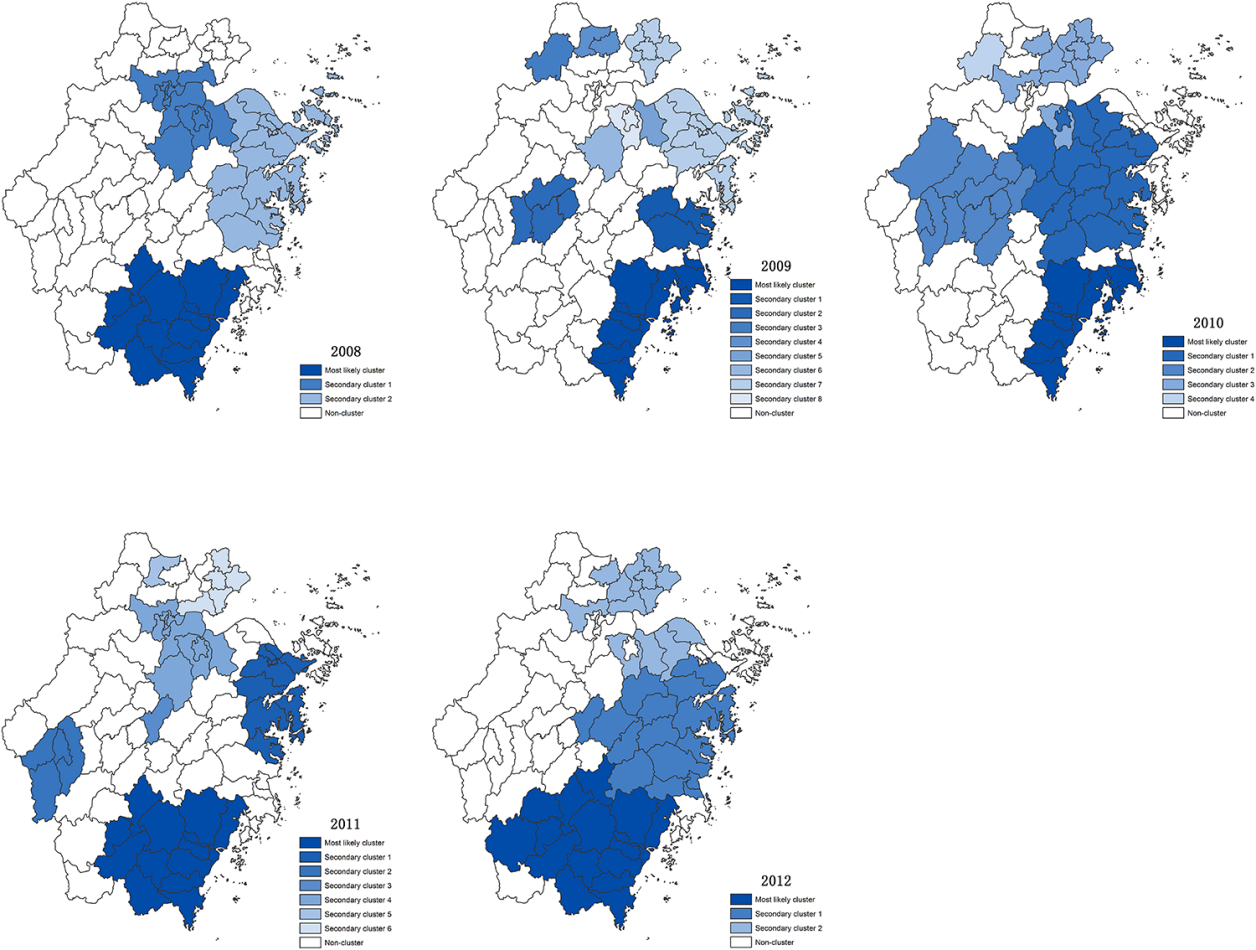
The results of space-time cluster analysis for mild cases of Zhejiang Province, 2008–2012.

Table 5 listed the scanning results of most likely clusters for mild cases derived from the retrospective space-time cluster analysis. The results showed that most likely clusters (dark blue) were mainly located at eastern coastal and southern regions (Fig 6). In 2008, 15 counties were classified into the most likely cluster (radius of the cluster was 93.26 km) from May to June where 7,807 cases were observed with a very high RR of 8.95 (*P*<0.001). During 2009 to 2012, RR was lowered and ranged from 3.74 to 4.36 while the number of the clusters was almost stable. EV71 was the major causative of the most likely clusters and the proportions of them were much higher than other regions (Table 5) which hinted that EV71 was associated with not only the severity of HFMD but also the clusters of HFMD. The second most likely clusters (light blue) illustrated in Fig 6 spread all over the province, especially those counties from Ningbo, Jinhua, Shaoxing and Quzhou. According to severe cases, the scanning results of most likely clusters were similar to those of spatial autocorrelation association and three districts (Wenzhou, Lishui and Ningbo) were major clusters (S2 Table and S8 Fig).

**Table 5:** The scanning results of space-time cluster analysis for mild cases of HFMD from Zhejiang Province, 2008–2012.

Year	Counties (n)	Radius (km)	Time (month)	Observed cases (n)	Expected cases (n)	Relative risk	*P*-value	Most likely clusters			Others		
								EV71 (%)	Cox A16 (%)	Others (%)	EV71 (%)	Cox A16 (%)	Others (%)
2008	15	93.26	5–6	7,807	1,075.81	8.95	<0.001	50.75	2.99	46.27	55.71	3.91	40.38
2009	12	85.41	9–12	16,102	4,594.96	4.26	<0.001	56.25	37.50	6.25	18.29	71.95	9.76
2010	12	85.41	4–7	26,086	7,415.45	4.27	<0.001	76.64	11.37	11.99	51.04	36.84	12.12
2011	14	93.26	5–7	15,272	4,049.33	4.36	<0.001	80.99	9.51	9.50	59.35	10.42	30.23
2012	17	98.60	4–6	23,981	7,273.37	3.74	<0.001	65.52	15.93	18.55	34.79	43.17	22.04

### Meteorological factors analysis

Since five districts (Wenzhou, Taizhou, Lishui, Ningbo and Quzhou) were the major clusters according to their incidences and the results of spatial autocorrelation analysis and space-time cluster analysis, we roughly defined them as clustered regions while other districts made up non-clustered regions. To explore the possible meteorological factors associated with the clusters of HFMD, the levels of monthly average temperature, monthly average rainfall and monthly average sunshine were compared between clustered regions and non-clustered regions during the peak of HFMD (April, May, June and July). Table 6 showed that sunshine was much lower in clustered regions than non-clustered regions during the peak period of the epidemics (three months had significant *P*-values of 0.032, <0.001 and 0.024, respectively using Student *t* tests and another month had a *P*-value of 0.064). Meanwhile no statistical differences were found on temperature and rainfall except rainfall in July (*P* = 0.003). Furthermore, S9 Fig detailed the rather lower levels of sunshine of clustered regions. As Wenzhou had a great epidemic of severe cases in 2010, we compared the levels of sunshine of Wenzhou since 2006. It was obvious that there was much less sunshine during the peak period in 2010 than other nearby years (S10 Fig).

**Table 6:** The comparison of meteorological factors between clustered regions and non-clustered regions during the peak period (April to July).

Factors	Month	Clustered regions (Mean ± SD)	Non-clustered regions (Mean ± SD)	*t*	*P*-values
Temperature (°C)	Apr	17.020±1.463	16.400±1.687	1.374	0.175
	May	21.835±0.857	21.814±1.150	0.070	0.944
	Jun	25.410±1.009	24.883±1.136	1.722	0.091
	Jul	29.255±0.722	29.423±1.037	-0.639	0.525
Rainfall (mm)	Apr	122.605±43.513	105.240±50.300	1.291	0.202
	May	135.165±90.277	112.309±60.067	1.127	0.265
	Jun	246.680±95.755	290.189 ±157.840	-1.272	0.209
	Jul	210.515±91.555	144.403±64.036	3.142	***0*.*003***
Sunshine (h)	Apr	140.040±33.303	161.826±36.260	-2.206	***0*.*032***
	May	152.900±37.241	192.706±37.093	-3.823	***<0*.*001***
	Jun	95.085 ±29.371	118.923±40.029	-2.326	***0*.*024***
	Jul	206.630±32.737	224.014±32.783	-1.893	0.064

## Discussion and Conclusions

Our study confirmed that Zhejiang Province was one of the highly epidemic provinces of HFMD with average annual incidence rate 172.98 per 100,000. Consistent with previous reports in China [4–6], children, especially boys under-five, were the most susceptible group. One of the important explanations was that elder children and adults may have been occult infected and developed antibodies to the enteroviruses. The seroprevalence summary provided the evidence that occult infection was common in elder children. The EV71 seroprevalence of the young children (0–5 years group) was about 29.1% while the elder children (6–10 years group and 11–20 years group) were 54.6% and 61.8%, respectively (Table 3 and Fig 5). In addition, the neonates’ high level of seroprevalence (44.0–75.0%) implied their mothers’ similar high level of antibody. Under such circumstance, children aged under-five may be the most susceptible group to HFMD. Furthermore, many studies reported that although elder children and adults had higher seroprevalence than younger children, their geometric mean titers (GMT) may be lower [31, 36, 42, 48]. It could be indirect evidence that enterovirus transmission may majorly occur in pre-school centers than at home [36, 42]. It was still unclear why boys had more chances to get infected. One possible explanation was that they usually spent more time on playing and were in close contact with their friends [25, 26]. Our EV71 seroprevalence survey confirmed that occult infection was common in children in Zhejiang Province. However, it was confusing that the elder children had more seroprevalence than the younger children (Table 2). Kuang et al. got the same results where ELISA was also used to detect EV71-IgG instead of neutralizing antibody [44]. ELISA was a fast and simple method for testing immunoglobulins such as IgG [61] and IgM [62, 63], which are serological markers of the past and ongoing infections. The relatively low level of GMT of elder children may decrease the sensitivity of ELISA which was an indirect method in nature [62, 63]. In addition, the fact that some subclasses of IgG did not have neutralizing activity may complicate the testing [61]. Therefore, neutralizing antibody should be recommended in the circumstances.

High-incidence clustered regions majorly consisted of the eastern coastal and southern counties (counties from Wenzhou, Taizhou, Lishui, Ningbo and Quzhou) whatever incidence rates comparison, spatial autocorrelation analysis or space-time cluster analysis was performed. One of the possible reasons is that the peaks of HFMD epidemics mostly occurred in April to July in Zhejiang Province due to the subtropical monsoon climate of warm weather, plentiful rainfall and low sunshine. Previously, researchers had found that the weather factors were associated with the incidence of HFMD [64, 65]. Furthermore, Deng et al. found that sunshine was negatively correlated with the incidence rate of HFMD [25] and Xing et al. associated the spring sunshine with the annual amplitude of epidemics [4]. We also showed that the clustered regions of Zhejiang Province had rather lower levels of sunshine than non-clustered regions (Table 6). Furthermore, Wenzhou, the district that had a great outbreak of severe cases in 2010, had quite low sunshine in that year according to the recent history (S10 Fig). Since the levels of other meteorological factors such as temperature and rainfall are similar in different regions of Zhejiang Province, sunshine may be the important meteorological factor for the reproduction or transmissibility of EV71 as well as for the early warning of epidemics of HFMD.

It was widely known that EV71 was the major cause of the severe cases of HFMD and our analysis found that the proportion of EV71 among all isolated viruses was correlated with the case-severity rate. When the proportions of EV71 reach 50% during April and July, the case-severity rate also reached their peaks for the year (Fig 4 and S5 Fig). Furthermore, the proportions of EV71 were much higher in the clustered regions than non-clustered regions (Table 5). Thus, the ecology of the enteroviruses may be important for the outbreaks of HFMD epidemics and the serological distribution of the enteroviruses can be a predictable factor for the early warning of epidemics of severe cases. In reality, the serotypes of the enteroviruses were measured only for the enteroviruses routinely isolated from most severe cases and a small part of mild cases. Therefore, it was an indirect index and biased estimate for investigating the serological distribution of the enteroviruses in the susceptible population and the clusters. Targeted sampling and serotyping may improve the accuracy of estimating the pathogens’ composition.

Phylogenetic analysis provides an essential way to track the variation and the evolution of the genomes of the enteroviruses and determine the phylogenetic relationships between the enteroviruses isolated from different regions. The genotypes of EV71 can be divided into A, B and C groups according to the phylogenetic tree of the VP1 gene [15]. Group B has five sublineages: B1–B5; and Group C also has five sublineages: C1–C5. C4 was originally detected in Japan in 1997 and then circulated in the Asia-Pacific regions, especially in mainland China [23, 66, 67]. Almost all EV71 strains isolated from China belonged to C4 while other genotypes such as B3, B4, B5, C3 and C5 had circulated in the Asia-Pacific region, and the exact reason is still unknown (S1 File and S11 Fig). From S11 Fig, we can conclude that the sequences of EV71 isolated from China were highly homologous and all Zhejiang strains originated from the country and were mixed with the strains from other provinces in the phylogenetic tree. Thus, the epidemics that occurred in Zhejiang Province were tightly related to those which occurred in other provinces. No sequence from Wenzhou was deposited in the NCBI database; however, Chen et al. reported that the VP1 sequences from Wenzhou (17 strains isolated during 2008–2010) were highly homologous to other Chinese strains and all belonged to the C4 group [68]. Therefore, no evidence showed that the Wenzhou epidemic of 2010 was caused by a novel EV71 strain and the high activity of EV71 (80% of pathogens isolated from Wenzhou were EV71) in that year can be a possible reason.

Since vaccines were still under phase III clinical trials [10, 11], the prevention and control of HFMD depended on interventions such as health education, regularly disinfection and minimizing patients’ public exposure. Our cluster analysis in this study was county-based, further subdistrict-based in the cities, and village-based in the counties, thus would support fine-mapping of the HFMD clusters. With these fine-mapping clusters, targeted interventions to HFMD could be performed more rapidly and efficiently. In the above discussion, evolutionary analysis has shown the phylogenetic relationships between the strains isolated from Zhejiang Province and the strains from other provinces, which has provided clues for the origin and the development of the enteroviruses. Cottam et al. [69] and Valdazo-Gonza´lez et al. [70] demonstrated that reconstructing of the transmission history of the foot-and-mouth disease virus using genomic sequences could support and help direct epidemiological investigations of the field outbreaks. Sequencing the genomes of the enteroviruses was not routine work even in the provincial CDCs in China today. We considered that routine sequencing and targeted sequencing for the outbreaks would be helpful for the tracking of the epidemics.

In summary, our study analyzed the epidemiological characteristics of HFMD in Zhejiang Province and revealed that the eastern costal and southern regions were the major locations of the clusters. We also found that pathogens’ serotypes and sunshine were the potential risk factors associated with these clusters. These findings can be helpful for the later prevention and control the HFMD epidemics in Zhejiang Province.

## Supporting Information

S1 Fig The incidence rates and case-severity rates of HFMD in Zhejiang Province, 2008–2012. (DOC)

S2 Fig The number of severe cases from Zhejiang Province (Wenzhou excluded), 2008–2012. (DOC)

S3 Fig The incidence rates of severe cases from Zhejiang Province, 2008–2012. (DOC)

S4 Fig The monthly distributions of pathogens’ serotypes (EV71, Cox A16 and other enteroviruses) of mild and severe cases from Zhejiang Province, 2008–2012. (DOC)

S5 Fig The partial correlations between the proportions of EV71/Cox A16 and the case-severity rate (Wenzhou excluded) from Zhejiang Province, 2009–2012. (DOC)

S6 Fig The LISA cluster map for mild cases from Zhejiang Province, 2008–2012. (DOC)

S7 Fig The LISA cluster map for severe cases from Zhejiang Province, 2008–2012. (DOC)

S8 Fig The results of space-time cluster analysis for severe cases from Zhejiang Province, 2008–2012. (DOC)

S9 Fig The monthly average sunshine (from April to July) of districts of Zhejiang Province, 2008–2012. (DOC)

S10 Fig The monthly average sunshine (from April to July) of Wenzhou during 2006–2012. (DOC)

S11 Fig Phylogenetic tree of the VP1 gene of EV71 strains isolated from China. (DOC)

S1 File Supplementary material and methods. (DOC)

S1 Table The Moran’s *I* of global spatial autocorrelation analysis for severe cases from Zhejiang Province, 2008–2012. (DOC)

S2 Table The scanning results of space-time cluster analysis for severe cases from Zhejiang Province, 2008–2012. (DOC)
