# Supplementary figures and images for: Epidemiological Characteristics and Spatial-Temporal Clusters of Hand, Foot, and Mouth Disease in Zhejiang Province, China, 2008-2012
Source: PLoS One. 2015 Sep 30;10(9):e0139109. doi: 10.1371/journal.pone.0139109 (PMC4589370; doi:10.1371/journal.pone.0139109)

**S1 Fig**. The incidence rates and case-severity ratesof HFMD in Zhejiang Province, 2008-2012.


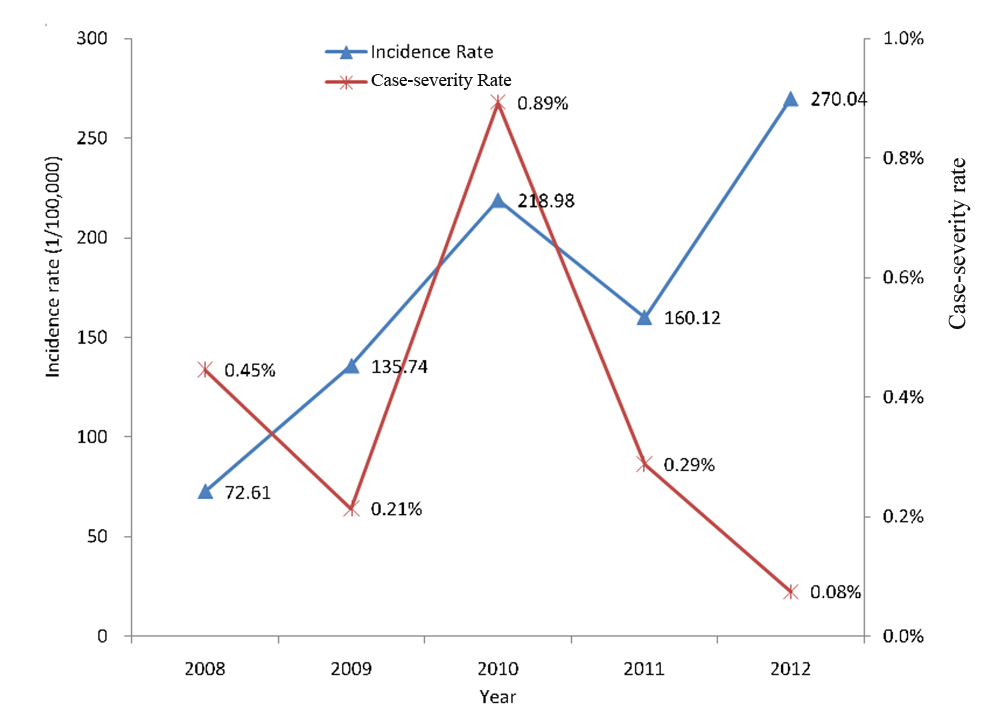

Supplement: S1 Fig — (DOC) [file pone.0139109.s001.doc]

**S2 Fig**. The number of severe cases from Zhejiang Province (Wenzhou excluded), 2008-2012.


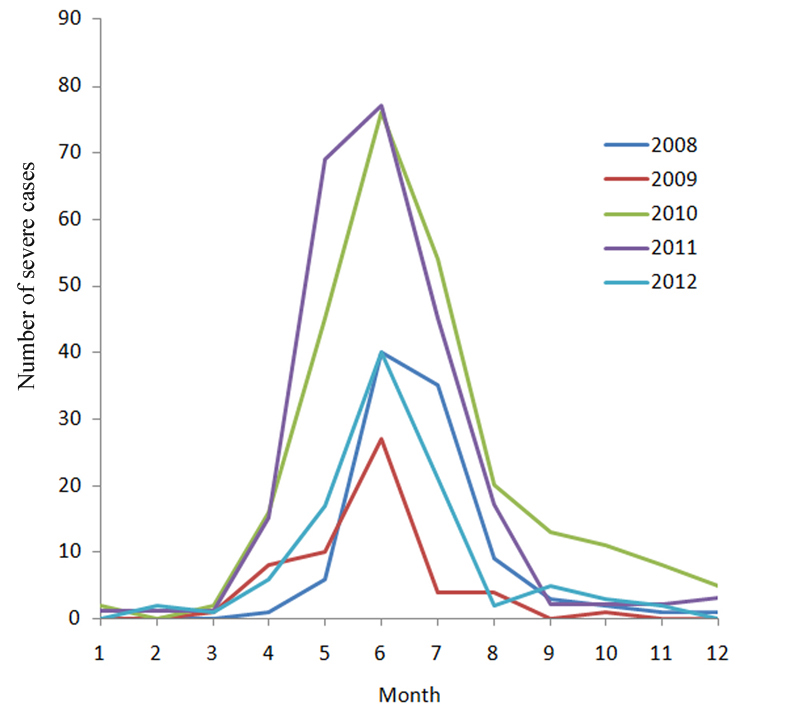

Supplement: S2 Fig — (DOC) [file pone.0139109.s002.doc]

**S3 Fig**. The incidence rates of severe cases from Zhejiang Province, 2008-2012.


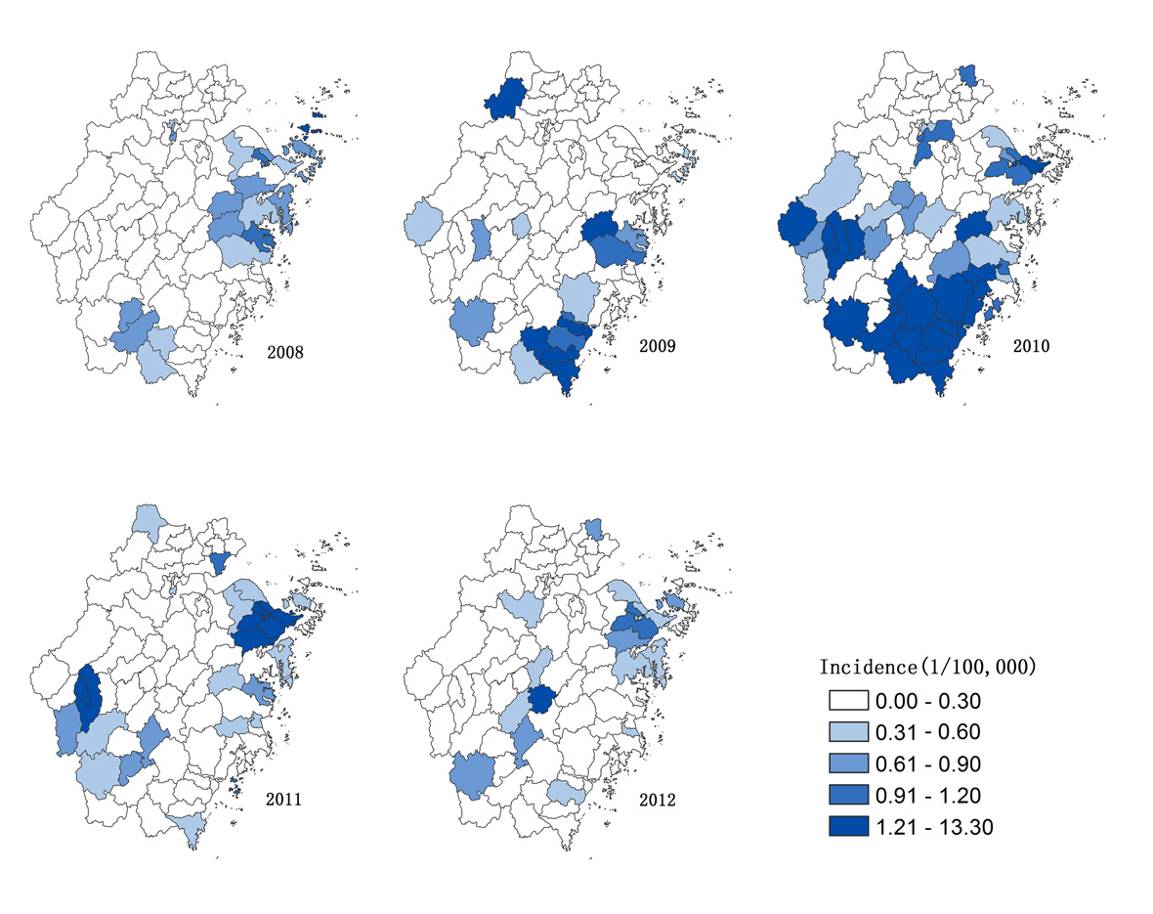

Supplement: S3 Fig — (DOC) [file pone.0139109.s003.doc]

**S6 Fig**. The LISA cluster map for mild cases from Zhejiang Province, 2008-2012.


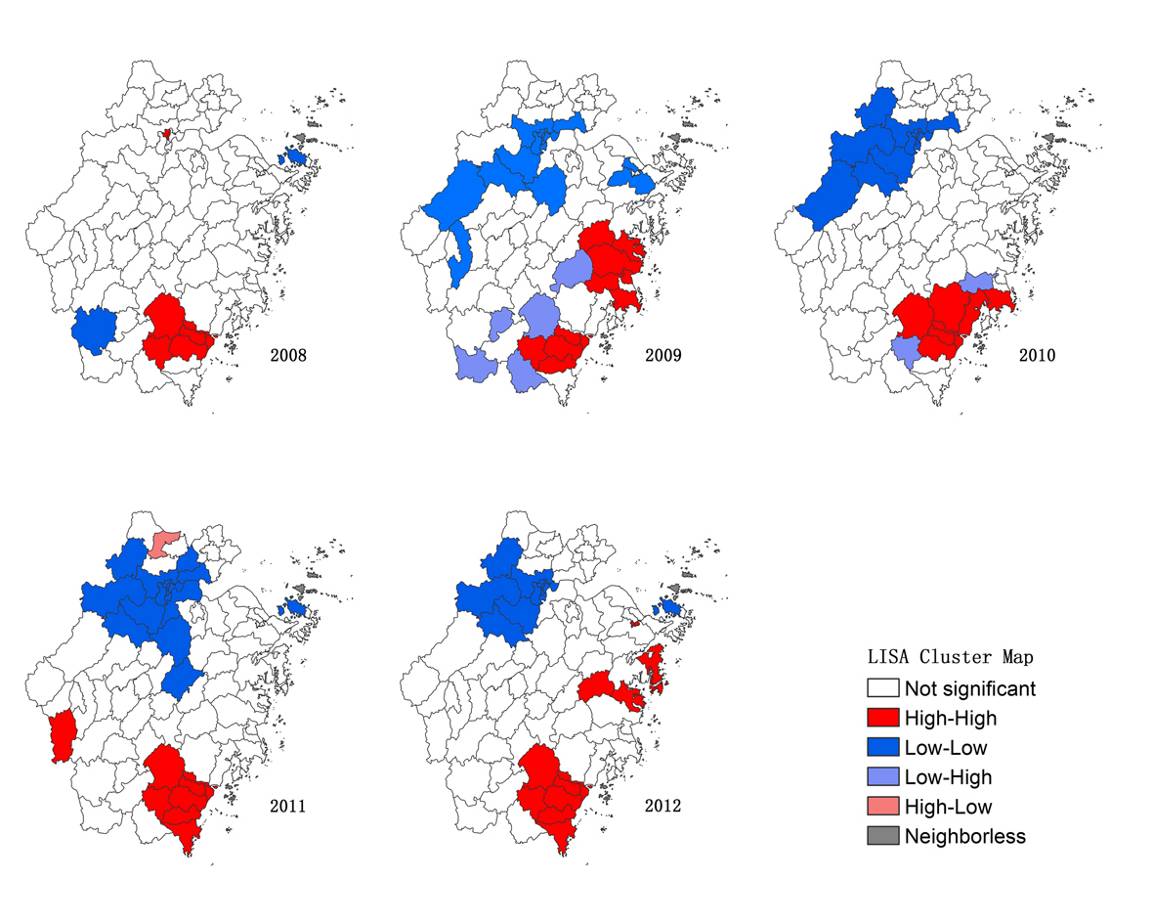

Supplement: S6 Fig — (DOC) [file pone.0139109.s006.doc]

**S7 Fig**. The LISA cluster map for severe cases from Zhejiang Province, 2008-2012.


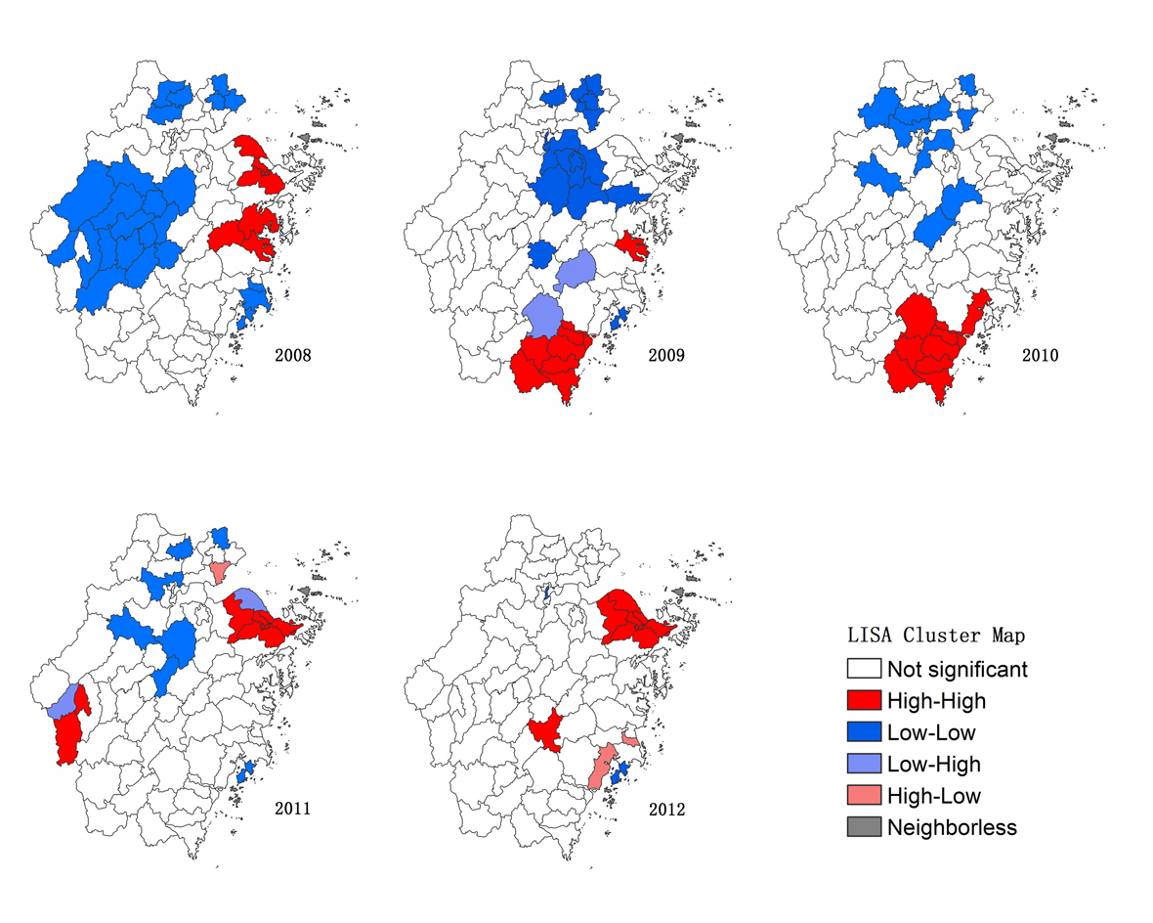

Supplement: S7 Fig — (DOC) [file pone.0139109.s007.doc]

**S8 Fig**. The results of space-time cluster analysis for severe cases from Zhejiang Province, 2008-2012.


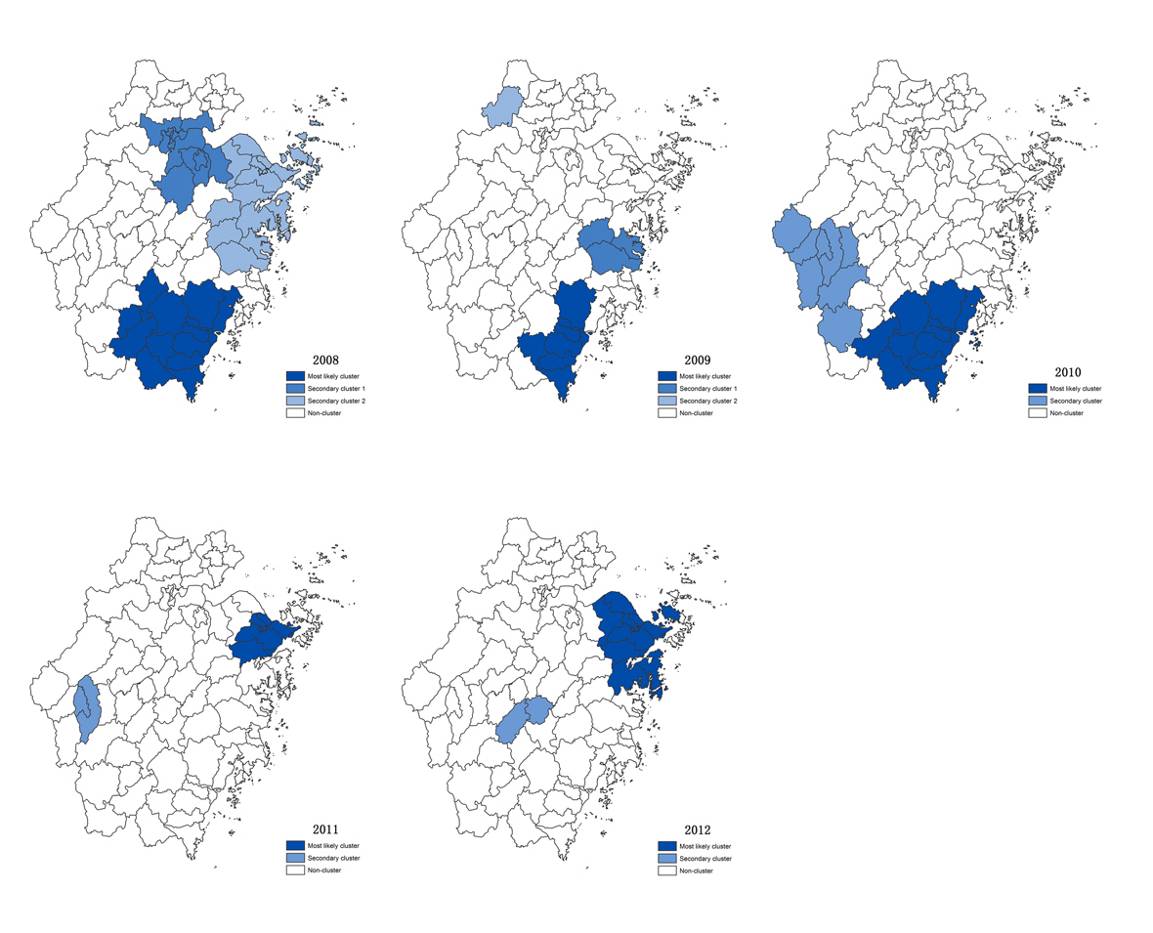

Supplement: S8 Fig — (DOC) [file pone.0139109.s008.doc]

**S9 Fig**. The monthly average sunshine (from April to July) of districts of Zhejiang Province, 2008-2012.


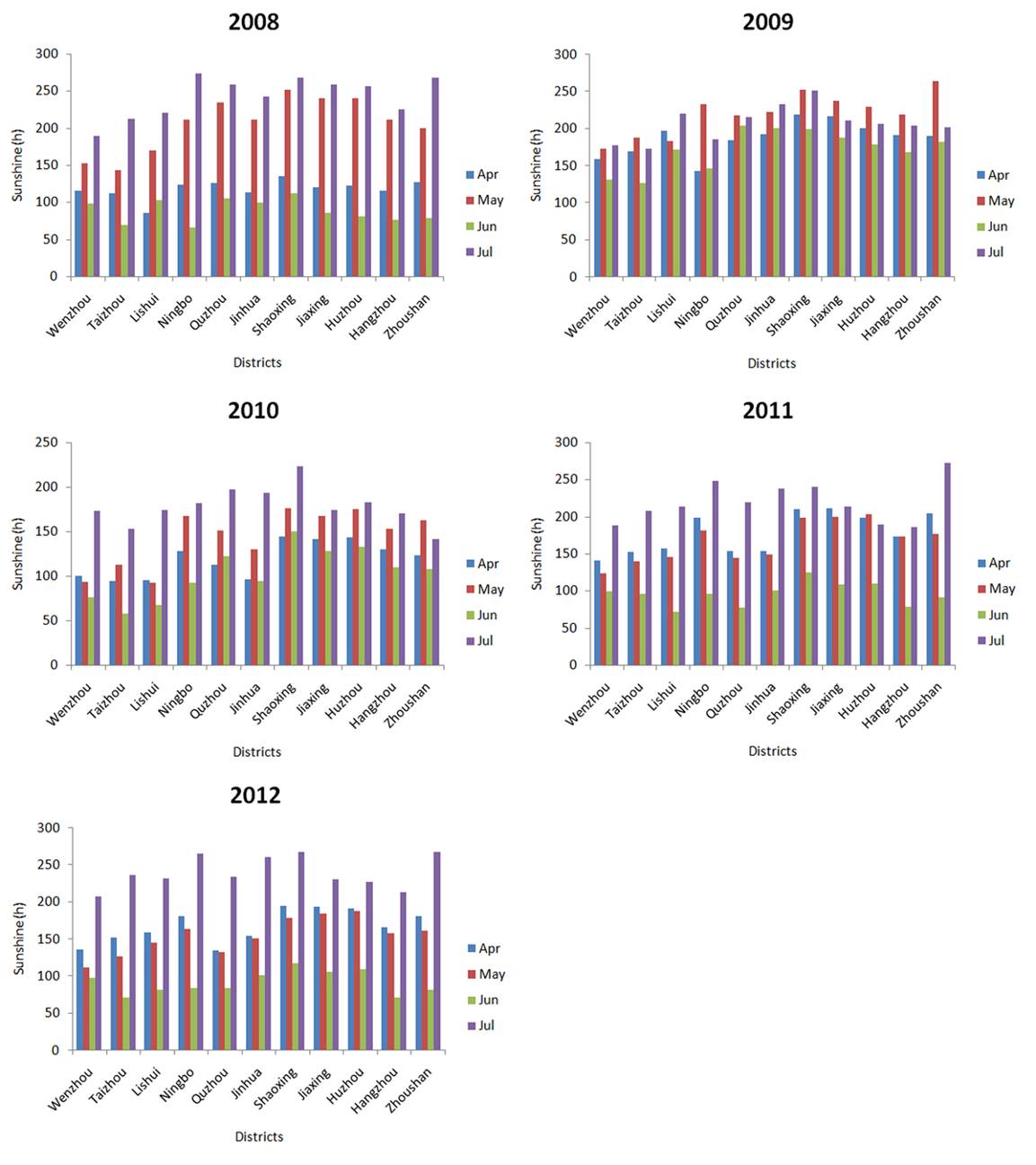

Supplement: S9 Fig — (DOC) [file pone.0139109.s009.doc]

**S10 Fig**. The monthly average sunshine (from April to July) of Wenzhou during 2006-2012.


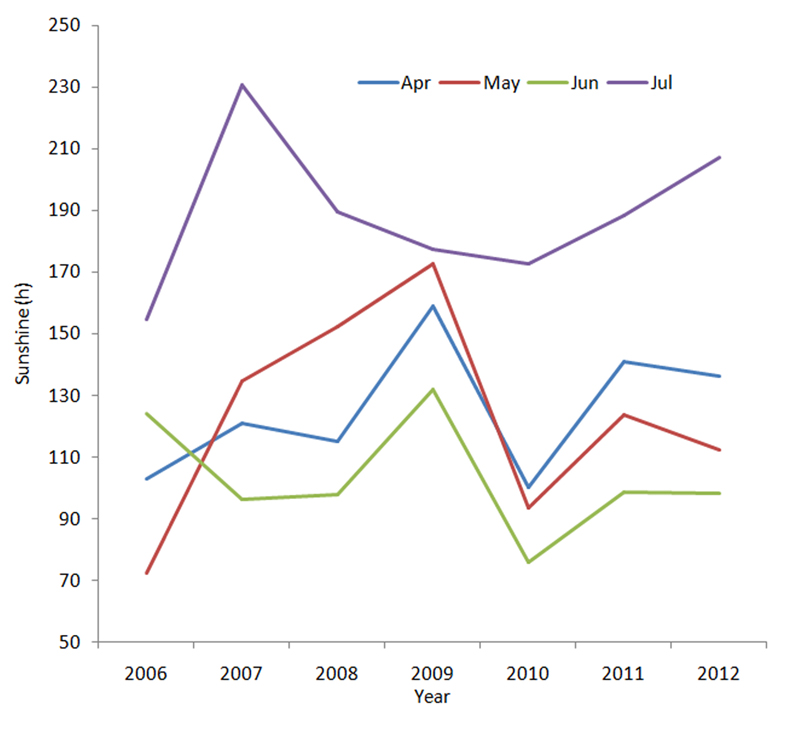

Supplement: S10 Fig — (DOC) [file pone.0139109.s010.doc]

**S11 Fig**. Phylogenetic tree of the VP1 gene of EV71 strains isolated from China.


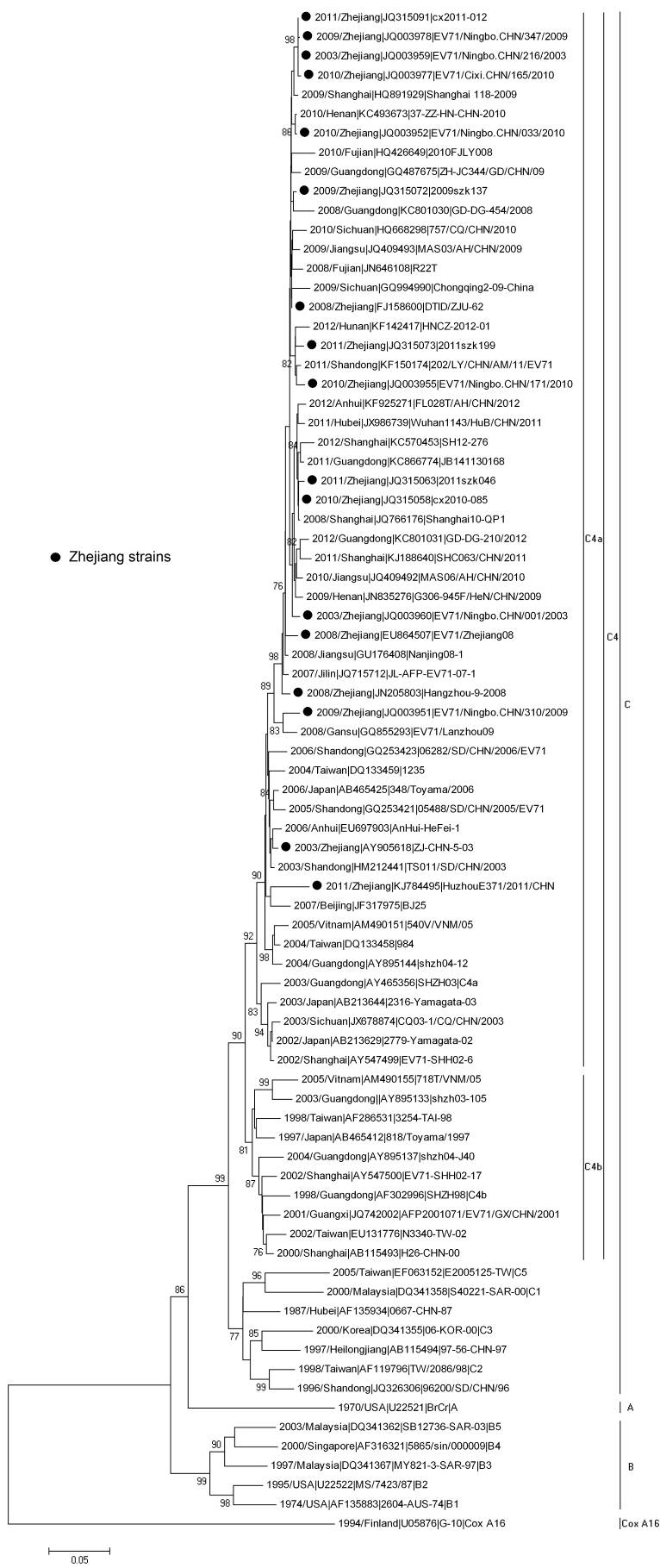

Supplement: S11 Fig — (DOC) [file pone.0139109.s011.doc]
