# Supplementary material for: Epidemiological Characteristics and Spatial-Temporal Clusters of Hand, Foot, and Mouth Disease in Zhejiang Province, China, 2008-2012
Source: PLoS One. 2015 Sep 30;10(9):e0139109. doi: 10.1371/journal.pone.0139109 (PMC4589370; doi:10.1371/journal.pone.0139109)

**S4 Fig**. The monthly distributions of pathogens’ serotypes (EV71, Cox A16 and other enteroviruses) of mild and severe cases from Zhejiang Province, 2008-2012.


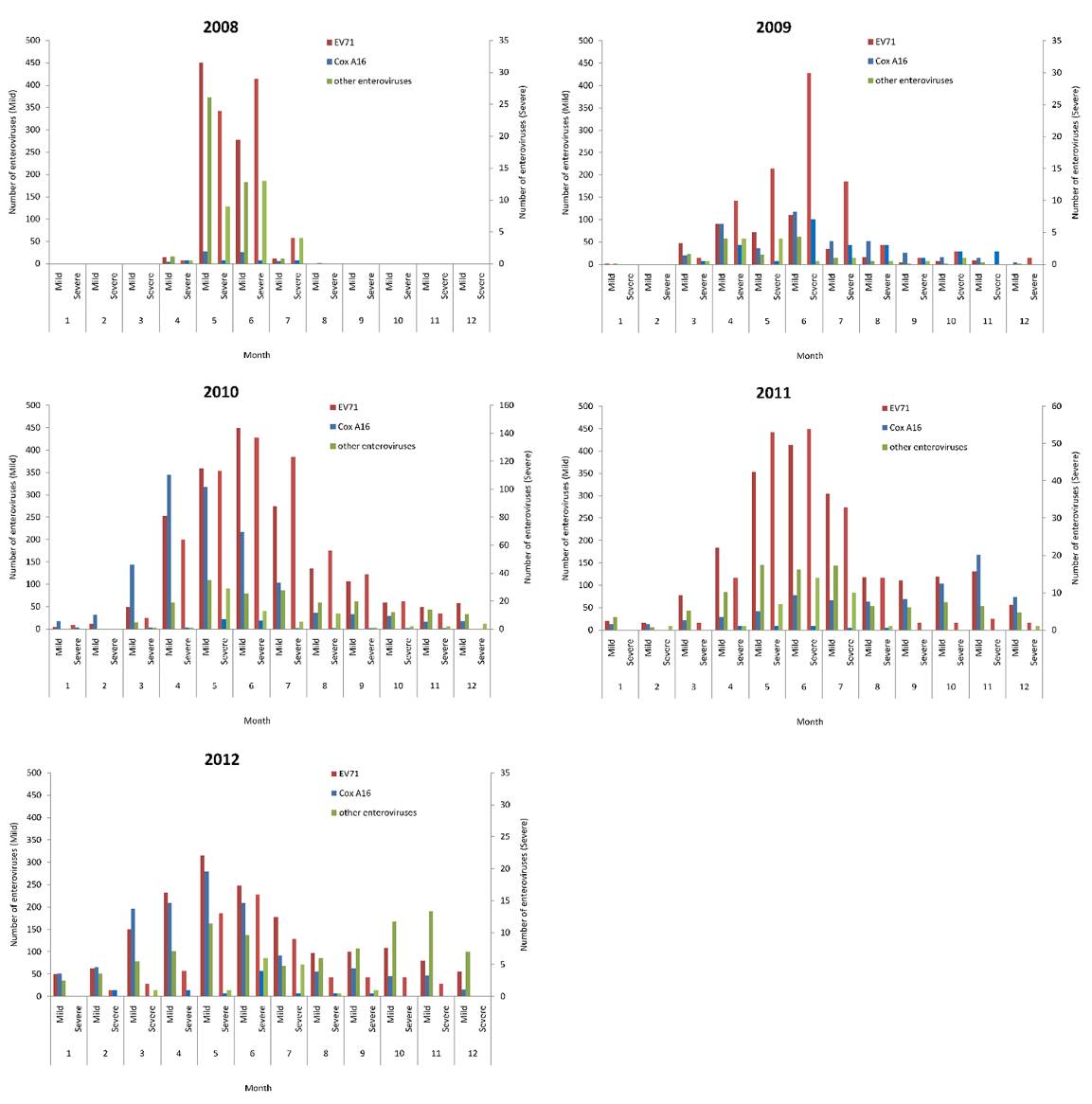

Supplement: S4 Fig — (DOC) [file pone.0139109.s004.doc]
