# Supplementary material for: Epidemiological Characteristics and Spatial-Temporal Clusters of Hand, Foot, and Mouth Disease in Zhejiang Province, China, 2008-2012
Source: PLoS One. 2015 Sep 30;10(9):e0139109. doi: 10.1371/journal.pone.0139109 (PMC4589370; doi:10.1371/journal.pone.0139109)
Supplement: S1 File — (DOC) [file pone.0139109.s012.doc]

**S1 File. Supplementary material and methods**

A phylogenetic tree for the VP1 gene of EV71 was constructed to show the phylogenetic relationships of EV71 strains isolated from China. A total of 953 VP1 sequences (strains were isolated between 1987 and 2012) and 20 sequences of reference strains (representing sublineages A, B1-B5, C1-C5) were downloaded from NCBI’s nucleotide database (http://www.ncbi.nlm.nih.gov/Taxonomy/Browser/

wwwtax.cgi?id=39054). Alignment of the sequences was performed based on the full coding region of VP1 (891 bps) using ClusterW implemented by Bioedit and sequences with lengths shorter than 891 bps were removed pre-alignment. Since most of the sequences were highly homologous, up to three Zhejiang strains and five strains for other provinces were randomly picked each year. In total, 58 Chinese strains (including 17 Zhejiang strains from 2003 to 2011) and 20 reference strains were used for the final alignment. A Coxsackievirus A16 (Cox A16) strain, G10 (1994/Finland/U05876), was used as an outgroup in the phylogenetic analysis. The phylogenetic tree was generated by the neighbor-joining method and bootstrap analysis was performed on 1,000 replicates. Only bootstrap values larger than 75% were shown on the phylogenetic tree.
