# Supplementary material for: Epidemiological Characteristics and Spatial-Temporal Clusters of Hand, Foot, and Mouth Disease in Zhejiang Province, China, 2008-2012
Source: PLoS One. 2015 Sep 30;10(9):e0139109. doi: 10.1371/journal.pone.0139109 (PMC4589370; doi:10.1371/journal.pone.0139109)
Supplement: S1 Table — (DOC) [file pone.0139109.s013.doc]

**S1 Table.** The Moran’s *I* of global spatial autocorrelation analysis for severe cases from Zhejiang Province, 2008-2012.

| Year | Moran's *I* | *Z* score | *P*-value |
| --- | --- | --- | --- |
| 2008 | 0.54 | 10.50 | <0.001 |
| 2009 | 0.43 | 7.12 | <0.001 |
| 2010 | 0.67 | 14.93 | <0.001 |
| 2011 | 0.49 | 8.42 | <0.001 |
| 2012 | 0.25 | 3.44 | <0.001 |
