# Supplementary material for: Epidemiological Characteristics and Spatial-Temporal Clusters of Hand, Foot, and Mouth Disease in Zhejiang Province, China, 2008-2012
Source: PLoS One. 2015 Sep 30;10(9):e0139109. doi: 10.1371/journal.pone.0139109 (PMC4589370; doi:10.1371/journal.pone.0139109)
Supplement: S2 Table — (DOC) [file pone.0139109.s014.doc]

**S2 Table.** The scanning results of space-time cluster analysis for severe cases from Zhejiang Province, 2008-2012.

| Year | Counties  (n) | Radius  (km) | Time  (month) | Observed  cases (n) | Expected cases (n) | Relative risk | *P*-value | Most likely clusters | | |  | Others | | |
| --- | --- | --- | --- | --- | --- | --- | --- | --- | --- | --- | --- | --- | --- | --- |
|  |  |  |  |  |  |  |  | EV71  (%) | Cox A16  (%) | Others  (%) |  | EV71  (%) | Cox A16  (%) | Others  (%) |
| 2008 | 21 | 160.70 | 5-8 | 100 | 9 | 25.35 | <0.001 | 40.35 | 0.88 | 58.77 |  | 57.61 | 4.75 | 37.64 |
| 2009 | 8 | 56.00 | 4-7 | 68 | 6.27 | 19.21 | <0.001 | 59.32 | 27.12 | 13.56 |  | 42.98 | 37.17 | 19.85 |
| 2010 | 13 | 92.69 | 4-9 | 727 | 84 | 28.18 | <0.001 | 80.15 | 5.76 | 14.09 |  | 50.63 | 35.49 | 13.83 |
| 2011 | 7 | 28.62 | 5-8 | 119 | 6.21 | 35.53 | <0.001 | 71.82 | 8.56 | 19.61 |  | 62.06 | 12.72 | 25.21 |
| 2012 | 12 | 64.57 | 6-7 | 38 | 2.72 | 20.82 | <0.001 | 30.29 | 36.54 | 33.17 |  | 50.72 | 29.88 | 19.40 |
